# The Zinc Finger Protein Zfr1p Is Localized Specifically to Conjugation Junction and Required for Sexual Development in *Tetrahymena thermophila*

**DOI:** 10.1371/journal.pone.0052799

**Published:** 2012-12-10

**Authors:** Jing Xu, Huaru Tian, Wei Wang, Aihua Liang

**Affiliations:** Key Laboratory of Chemical Biology and Molecular Engineering of Ministry of Education, Institute of Biotechnology, Shanxi University, Taiyuan, China; Nanjing Medical University, China

## Abstract

Conjugation in *Tetrahymena thermophila* involves a developmental program consisting of three prezygotic nuclear divisions, pronuclear exchange and fusion, and postzygotic and exconjugant stages. The conjugation junction structure appears during the initiation of conjugation development, and disappears during the exconjugant stage. Many structural and functional proteins are involved in the establishment and maintenance of the junction structure in *T. thermophila*. In the present study, a zinc finger protein-encoding gene *ZFR1* was found to be expressed specifically during conjugation and to localize specifically to the conjugation junction region. Truncated Zfr1p localized at the plasma membrane in ordered arrays and decorated Golgi apparatus located adjacent to basal body. The N-terminal zinc finger and C-terminal hydrophobic domains of Zfr1p were found to be required for its specific conjugation junction localization. Conjugation development of *ZFR1* somatic knockout cells was aborted at the pronuclear exchange and fusion conjugation stages. Furthermore, Zfr1p was found to be important for conjugation junction stability during the prezygotic nuclear division stage. Taken together, our data reveal that Zfr1p is required for the stability and integrity of the conjugation junction structure and essential for the sexual life cycle of the *Tetrahymena* cell.

## Introduction

Cell junctions provide contact between neighboring cells or between a cell and the extracellular matrix in multicellular organisms. Intercellular junctions serve to maintain cell and tissue polarity and integrity, integrate intra- and intercellular signaling. In vertebrates, there are three major types of cell junctions, namely, anchoring, occluding, and communicating junctions. Anchoring junctions mechanically attach cells to their neighbors or to the extracellular matrix and organize the cortical cytoskeleton beneath the plasma membrane to modulate cell and tissue behavior [1]. Occluding junctions seal cells together in the epithelium in a way that prevents even small molecules from passing from one side of the sheet to the other [2], [3]. Communicating junctions are aqueous intercellular channels that allow the diffusion of small molecules and ions from cell to cell [4]. The molecules responsible for creating cell junctions include various cell adhesion molecules, such as selectins, cadherins, integrins, and members of the immunoglobulin superfamily [5]. These core junctional components are also assisted by additional cell-type specific and accessory molecules, which cooperate to tailor junctions structurally and functionally [1].

In unicelluar protists, intercellular conjugation junctions are required for sexual reproduction. Conjugation is a highly conserved developmental process in ciliates that has been well studied [6], [7]. In preparation for conjugation, *Tetrahymena* cells actively modify their pattern of protein synthesis. The anterior ends of the cells transform from a pointed to a blunt shape and from ciliated and ridged to smooth in texture [8]. During the course of conjugation, the conjugation junction undergoes dramatic membranous metamorphosis [8], [9]. A number of cisternae are present between the junction and kinetosomes of the adoral zone of the membranelles [10], [11]. In *Tetrahymena*, a cell–cell junction is robust in order to survive the mechanical stresses experienced when two individual and highly mobile cells attempt to form a union [12]. An elaborate conjugation junction structure is also required for pronuclear exchange during the conjugation stage [13]. The pronuclear exchange is impelled by microtubule-rich baskets whose terminals are connected with the junction. After a reciprocal nuclear exchange, the junction reestablishes integrity [10]. Using a proteomics-based approach, 15 proteins in the *Tetrahymena* conjugation junction structure have been identified. They include fenetrin, several cytoskeletal, nuclear, mitochondrial, ribosomal proteins and hypothetical proteins [12]. Fenetrin is a structural protein, it could facilitate the events surrounding the exchange of genetic material at the mating junction, by providing a structural scaffold at the junction between mating cells [12]. Recently, it has been reported that Cda13p associated with membrane trafficking is transiently localized on the resealed conjugation junction and participates in events associated with remodeling of the nuclear exchange junction during conjugation [14]. Although some conjugation junction structural proteins have been identified in *Tetrahymena*, the molecular mechanism underlying the process remains poorly understood.

Zinc finger proteins are among the most abundant proteins in eukaryotic genomes. Their functions are extraordinarily diverse and include DNA recognition, protein folding and assembly, protein–protein interactions as well as membrane association [15]. In higher eukaryotes, the zinc finger protein ZFPL1 is a conserved and widely expressed integral membrane protein with two predicted zinc fingers at the N-terminus. ZFPL1 interacts with the cis-Golgi matrix protein GM130 and is important for the integrity of cis-Golgi [16]. During *C. elegans* spermatogenesis, the zinc finger containing protein spe-10 is required for membranous organelles to properly deliver lysosome-related fibrous bodies to the *C. elegans* spermatid and its DHHC–CRD zinc finger motif is essential for this function [17]. Based on the published macronucleus genome and the gene expression profiles of *T. thermophila* [18], [19], more than two hundred zinc finger domain containing genes have been predicted (http://www.ciliate.org; http://tfgd.ihb.ac.cn). One such zinc finger domain containing gene named *ZFR1* (zinc finger-related protein, TTHERM_01285910) was identified in the present study. Immunofluorescence staining for Zfr1p showed that it was specifically localized in the conjugation junction structure. Deletion of its zinc finger and hydrophobic domains altered its localization. Truncated Zfr1p decorated foci located adjacent to the basal body. This localization pattern is consistent with the location of the Golgi apparatus, which is localized predominantly in the cell cortex and is closely associated with the mitochondria [20]. The mating pair of a *ZFR1* knockout cell was less stable than that of a wild-type and *ZFR1* overexpressing cell. Conjugation development of a somatic *ZFR1* knockout cell was aborted 8–10 h into the mating stage, possibly due to defects in pronuclear exchange or reestablishment of conjugation junctions. These results showed that Zfr1p is required for the stability and integrity of conjugation junctions in *Tetrahymena*. Furthermore, Zfr1p is essential for the sexual life cycle of *Tetrahymena*.

## Materials and Methods

### *Tetrahymena* strains and culture conditions

The wild-type B2086 (mating type II) and CU428 (micronuclear genotype Mpr/Mpr; mating type VII) strains of *T. thermophila* were provided by Dr. Peter. J. Bruns (Cornell University,Ithaca, NY, now available through the National Tetrahymena Stock Center, http://tetrahymena.vet.cornell.edu/index.html). The cells were grown in SPP medium [21] at 30°C. For analysis of conjugation, log-phase growing cells of different mating types were washed, starved (16–24 h at 30°C), and mixed in 10 mM Tris-HCl (pH 7.4) at equal amounts (∼2×10^5^ cells/ml), as previously described [22].

### Cloning of the *ZFR1* gene

The patterns of gene expression during conjugation correlate well with the developmental stages of meiosis, nuclear differentiation and DNA elimination. *EZL1*, which is expressed specifically during conjugation stage, catalyzes scnRNA-dependent K27 methylation and is required for internal eliminated sequences elimination [23]. A total of 51 genes were found to be coexpressed with *EZL1* [19]. One of the genes, TTHERM_01285910, which we chose to study and named *ZFR1*, was found to be co-expressed with *EZL1* with a correlation coefficient of 0.997. The *ZFR1* gene was first identified from the *T. thermophila* database (http://www.ciliate.org). The expression profile of this gene was also obtained from the *T. thermophila* microarray database (http://tfgd.ihb.ac.cn) [19]. Total RNA was extracted from *Tetrahymena* cells using Trizol (Takara) and was treated with RNase-free DNase I (Takara). The first-strand cDNA was synthesized using a PrimeScript^TM^ reverse transcriptase (RT) and random hexamer primers. The *ZFR1* cDNA was cloned and sequenced. Expression profile of *ZFR1* was then confirmed by quantitative RT polymerase chain reaction (qRT-PCR). qRT-PCR was performed with the SYBR Premix Ex Taq^TM^ (Takara) on a ABI StepOne Plus system (Applied Biosystems, USA). Each reaction was performed in triplicate. The values were normalized to the expression of the ribosomal 17S rRNA as an internal control. The primers qRT-Pup and qRT-Pdown were used (Table S1). The following parameters were used for PCR: 10 min at 95°C, followed by 40 cycles of 95°C for 15 s, 53°C for 30 s, and 68°C for 35 s. A melting curve of the PCR products (60–90°C) was also obtained to ensure the absence of artifacts.

### Construction of somatic knockout *ZFR1* strains

To create the targeting construct, the 5′ and 3′ flanking regions of *ZFR1* were amplified from genomic DNA using the PCR primers KO-5′ FW and KO-5′ RV, and KO-3′ FW and KO-3′ RV (Table S1), respectively. The *neo4* cassette conferring paromomycin sulfate resistance was amplified using primers *neo4*FW and *neo4*RV (Table S1). The *ZFR1* knockout construct was obtained by overlapping PCR using primers KO-FW and KO-RV (Table S1). B2086 and CU428 cells were transformed with the respective constructs using the Biolistic PDS-1000/He particle-delivery system (Bio-Rad), as previously described [24]. Transformants were selected on the basis of resistance to paromomycin. To obtain somatic *ZFR1* knockout strains, the cells were subjected to stepwise selection in increasing concentrations of paromomycin sulfate in the presence of 0.1 µg/ml CdCl_2_, starting from 60 μg/ml to a final concentration of 50 mg/ml until the cells failed to grow. The endogenous macronuclear *ZFR1* gene was completely replaced by phenotypic assortment and selection in increasing concentrations of paromomycin sulfate. (The genotypes and phenotypes of all the strains used in this study are provided in Table S2).

### Viability test

After 5–6 h of mating, 300 individual *ZFR1* knockout cells and WT mating pairs were isolated and incubated in single SPP medium drops, as previously described [25], [26]. Three hours later, the drops were examined to exclude cells that had been killed during pair isolation. After 48 h, the drops were re-examined. To check for complete conjugation in wild-type (B2086× CU428) and *ZFR1* knockout cells (△*ZFR1*-B2× △*ZFR1*-C4), the cells were incubated using 15 µg/ml 6-methylpurine (6-mp; Sigma) or 60 µg/ml paromomycin sulfate in SPP.

### Construction of HA-*ZFR1* strains

To create the hemagglutinin (HA)-*ZFR1* construct, the HA coding sequence was inserted next to the initiation codon of *ZFR1* by overlapping PCR. The primers used for the PCR were HA-5′FW and HA-5′RV, HA-3′FW and HA-3′RV, and HA-*neo4*FW and HA-NextRV (Table S1). The *Neo4* cassette [27] was introduced into the 5′ flanking sequence of HA-*ZFR1* by PCR. The primers used for PCR were HA-*neo4*FW and HA-*neo4*RV, and *neo4*-5′FW and *neo4*-3′RV. The HA-*ZFR1-neo4* construct was obtained by overlapping PCR with the primers HA-FW and HA-RV. CU428 and B2086 cells were transformed with the respective constructs using the Biolistic PDS-1000/He particle-delivery system (Bio-Rad Laboratories, USA), as previously described [24]. The endogenous macronuclear *ZFR1* gene was completely replaced by phenotypic assortment and selection in increasing concentrations of paromomycin sulfate.

### Western blot analysis

Whole-cell proteins from 2.0×10^3^ cells were separated by sodium dodecyl sulfate polyacrylamide gel electrophoresis, and transferred onto polyvinylidene fluoride membranes. The blots were incubated with 1∶2000 diluted mouse anti-HA antibodies (16B12; Covance, Berkeley, CA) in a blocking solution (1% bovine serum albumin, 1% nonfat dry milk, and 0.1% Tween 20 in phosphate-buffered saline), followed by a 1∶10000 dilution of horseradish peroxidase-conjugated goat anti-mouse immunoglobulin G (IgG) (Zymed Laboratories Inc., South San Francisco, CA) in a blocking solution. The membranes were then washed four times in 10 mM Tris-buffered saline with 0.1% Tween 20. The bound antibodies were visualized using enhanced chemiluminescence reagents (PerkinElmer Life Sciences, Boston, MA, USA) [26].

### Indirect immunofluorescence staining

The cells were fixed overnight using Lavdowsky's fixative (ethanol/37% formaldehyde/acetic acid/water; 50∶10∶1∶39) at 4°C, and immobilized on cover glasses coated with poly-L-lysine (Sigma). The samples were incubated with 1∶200 dilution of anti-HA antibodies (California Bioscience, Coachella, CA, USA) in blocking solution, followed by 1∶200 dilution of fluorescein isothiocyanate-conjugated anti-mouse IgG ( Zymed Laboratories, Carlsbad, CA, USA) in blocking solution. The samples were incubated with 1 μg/ml 4′,6-diamidino-2-phenylindole dihydrochloride (DAPI) (Roche Diagnostics, USA) in PBS (137 mM NaCl, 2.7 mM KCl, 10 mM Na_2_HPO_4_, 2 mM KH_2_PO_4_, pH = 7.4), mounted, and observed using an Olympus BH-2 or FV1000 fluorescence microscope (Tokyo, Japan).

For dual staining of basal body/HA-Zfr1p, cells were fixed in Lavdowsky's fixative as described above. Basal bodies were visualized using 1∶200 dilution of the monoclonal antibody 20H5 (Cat. No. 04-1624) that recognizes centrin and 1∶100 dilution of TRITC-conjugated donkey anti-Mouse antibody (Cat. No. AP192R). Similarly, HA-Zfr1p was visualized using 1∶200 dilution of rabbit monoclonal HA antibody (Cat. No. 05-902R) and 1∶100 dilution of FITC-conjugated goat anti-rabbit IgG antibody (Cat. No. AQ132F) (All Millipore, GmbH Schwalbach/Ts, Germany). Nuclei were stained with 1 µg/mL DAPI (Roche Diagnostics, USA). Cells were imaged using the DeltaVision imaging system (Applied Precision, Inc., WA, USA). The images were adjusted for contrast and brightness using Adobe Photoshop CS (Adobe, San Jose, CA).

### Construction of HA-*ZFR1* overexpression strains

To generate the HA-*ZFR1* overexpression construct, *ZFR1* cDNA was first amplified by PCR using the primers OE-FW and OE-RV (Table S1), then inserted into the pBS-HA plasmid in such a way that expression of HA-*ZFR1* was controlled by the *MTT1* promoter. pBS-HA-*ZFR1* was digested with *Sac*I, and introduced into the CU428 and B2086 strains using the Biolistic PDS-1000/He particle-delivery system (Bio-Rad). The transformants were selected based on paromomycin resistance. The MTT1 replacement was analyzed by PCR with a pair of specific primers (*MTT1-*FW and *MTT1-*RV) (Table S1). Then, HA-*ZFR1* over-expression was analyzed by real-time quantitative PCR using primers qRT-Pup and qRT-Pdown (Table S1). The following parameters were used for the PCR: 10 min at 95°C, followed by 40 cycles of 95°C for 15 s, 53 °C for 30 s, and 68°C for 35 s. A melting curve of the PCR products (60–90°C) was also obtained to ensure absence of artifacts. The values were normalized to the expression of the ribosomal 17S rRNA as an internal control.

### Conjugation junction stability assay

After 3 h of mating, 200 µl of mating cells of wild type and *ZFR1* knockout strains were collected and placed in 1.5 ml Eppendorf tubes. The samples were shaken on a vibrator at 8000 rpm for 5 min. Then, 20 µl of 20% trichloroacetic acid was added, and the numbers of paired and single cells were counted. Sampling of the cells was repeated three times.

### Construction of truncated *ZFR1* strains

The 5′-truncated-*ZFR1*, 3′-truncated-*ZFR1*, and 3′, 5′-truncated-*ZFR1* genes were amplified by PCR using the specific primers truncated-5′-FW and truncated-5′-RV, truncated-3′-FW and truncated-3′-RV, and 5′*BamH*1-T-FW and 3′*Asc*1-T-RV (Table S1), respectively. The PCR products were then digested with *BamH*1 and *Asc*1 before cloning into a pBS-HA vector digested with *BamH*1 and *Asc*1. CU428 and B2086 cells were transformed with these constructs using the Biolistic PDS-1000/He particle-delivery system (Bio-Rad Laboratories, USA).

### Brefeldin A

Brefeldin A was added to cells at a concentration of 10 µg/ml. Cultures were incubated at 30°C for 3 h and CdCl_2_ was added at 0.2 ug/ml after 2 h. Then, cells were starved and mated. Mating cells at 6 h postmixing were collected and examined for the localization of HA-tagged Zfr1p [14].

## Results

### Characterization of *ZFR1*

**Figure 1:**
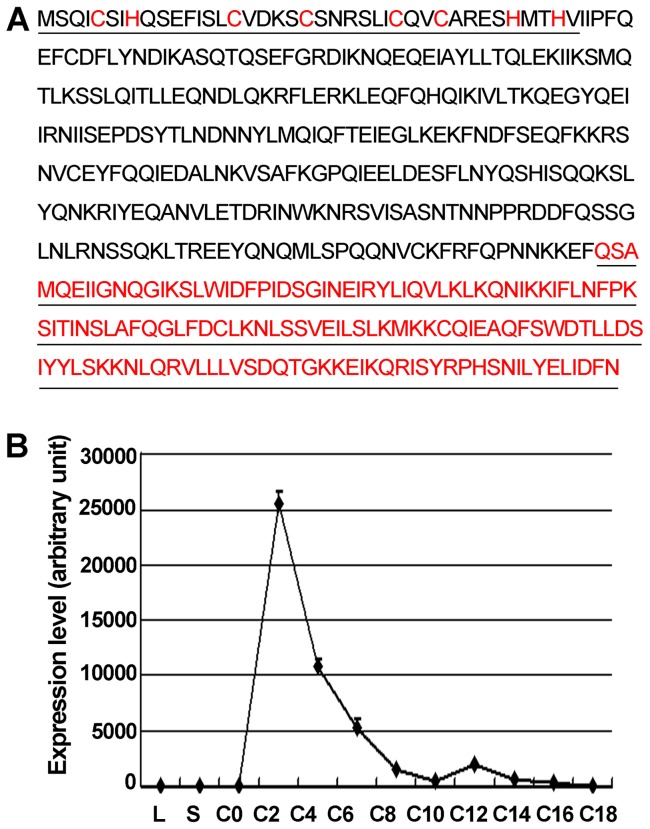
Characterization of *ZFR1.* (A) Analysis of the amino acid sequence of *ZFR1*. Bold letters indicate the N-terminal zinc finger domain of Zfrp1, and underlined letters indicate the C-terminal hydrophobic domain of Zfr1p. (B) qRT-PCR analysis of *ZFR1* expression profile. Y-axis indicates relative fluorescence strength. X-axis indicates developmental stages of the strains. Total RNA from log-phase growing (L), starved (S), and conjugating cells (C-0, 0 hr; C-2, 2 hr; C-4, 4 hr; C-6, 6 hr; C-8, 8 hr; C-10, 10 hr; C-12, 12 hr; C-14, 14 hr; C-16, 16 hr; C-18, 18 hr).

*ZFR1* was first identified in the *Tetrahymena* macronuclear genome database (http://www.ciliate.org), confirmed by PCR, then sequenced (data not shown). *ZFR1* is 1347 bp long and consists of four exons that encode a predicted protein of 448 amino acids. The deduced protein sequence of Zfr1p contains an N-terminal B-Box zinc finger domain, and a C-terminal hydrophobic region (Fig. 1A). qRT-PCR analysis showed that *ZFR1* was not expressed during the vegetative growth and starvation stages but was expressed specifically during the conjugation stages. In particular, the expression was up-regulated 2 h into the conjugation stage (Fig. 1B). This result is consistent with the expression profile of *ZFR1* revealed by microarray data (Fig. S1) [19]. *ZFR1* mRNA abundance was found to peak during the conjugation stage, implying that *ZFR1* may play an important role in the process.

It is well known that genes encoding proteins known to interact or to function in complexes show similar expression patterns, co-ordinate expression with putative genes of known function can identify genes with related functions [19]. To identify the signalling pathway by which Zfr1p functions, four genes co-expressed with Zfr1p were identified using the TGED database (http://tged.ihb.ac.cn/search.aspx?keyword  = TTHERM_01285910) (Fig. S1) [19]. Of those, we found that *TLR1* (TTHERM_00408910 encodes a transmembrane protein and *TDT1* (TTHERM_00335970) encodes delta tubulin (correlation coefficient of co-expression with *ZFR1* R = 0.987 and R = 0.948, respectively). Moreover, two new zinc finger domain containing genes *ZFR2* (TTHERM_00637350) and *ZFR3* (TTHERM_00531890) (correlation coefficient of co-expression with *ZFR1* R = 0.999,0.869,respectively) were also identified. A gene network is useful to identify the genes involved in the same pathway, in a protein complex or that are co-regulated. Furthermore, Tetrahymena gene network (TGN) also indicated that *ZFR1* is co-expressed with *TLR1, TDT1, ZFR2* and *ZFR3* [28].

### *ZFR1* is not essential in vegetative cells

**Figure 2:**
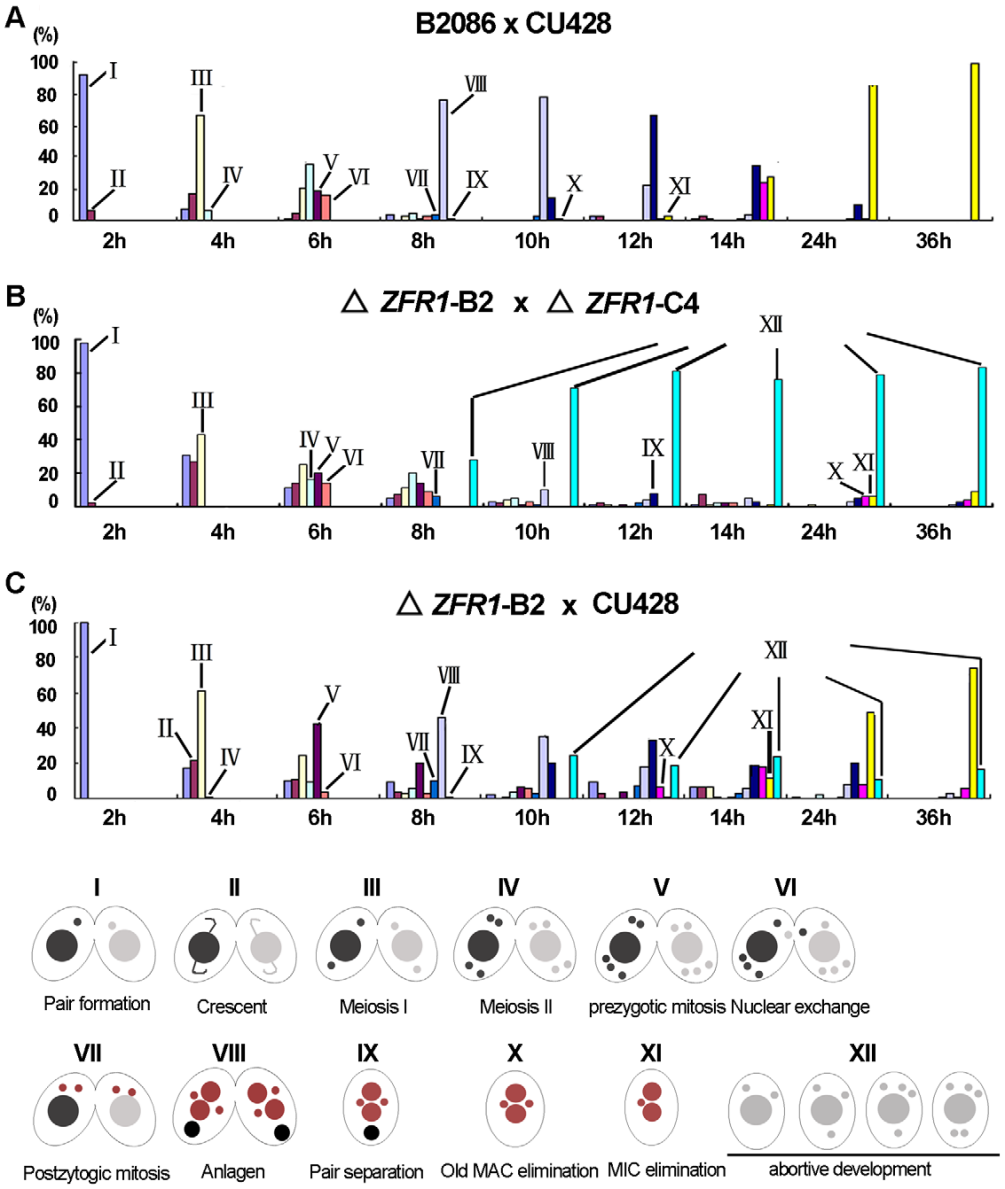
Somatic knockout of *ZFR1.* (A) Schematic illustrating generation of the *ZFR1* knockout strain. A drug resistance marker (*neo4*) was recombined into the *ZFR1* gene locus and this replaced the endogenous *ZFR1* coding sequence. Arrows indicate the positions of primers used for PCR. (B) Analysis of *ZFR1* knockout strains. Five somatic knockout *ZFR1* heterozygotic strains and 1 wild-type strain were identified by PCR. PCR fragments indicated that the *ZFR1* macronuclear copy was completely replaced in the Δ*ZFR1-B* and Δ*ZFR1-C* strains. (C) Confirmation of *ZFR1* knockout by qRT-PCR. Y-axis indicates relative fluorescence strength. X-axis indicates conjugation stages of the strains. Wild-type cells and *ZFR1* knockout cells were mated. RNAs were isolated 2, 4, 6, and 8 h after mixing.

To study the function of *ZFR1*, the open reading frame (ORF) of *ZFR1* in the polyploid macronucleus was replaced by paromomycin resistance gene by homologous recombination (Fig. 2A). Replacement of the *ZFR1* ORF was verified by PCR in five independent somatic knockout strains using a pair of specific primers (*ZFR1*-Fi and *ZFR1*-Ri). A single band, whose size was consistent with the size of the Neo4 cassette fragment, was amplified (Fig. 2B). To further confirm *ZFR1* complete somatic knockout of *ZFR1*, different *ZFR1* knockout cells, namely, △*ZFR1*-C4 and △*ZFR1*-B2, were mated. qRT-PCR analysis showed that no *ZFR1* transcripts were produced before new macronuclei formed, but *ZFR1* expression was restored when the new macronucleus formed after 8 h postmixing (Fig. 2C). This result showed that the parentally somatic macronuclear *ZFR1* gene was completely replaced. The *ZFR1* somatic knockout strains showed no obvious defects during vegetative growth (data not shown). This result was expected since *ZFR1* is not expressed in growing and starved cells.

### Zfr1p is required for conjugation development

**Figure 3:**
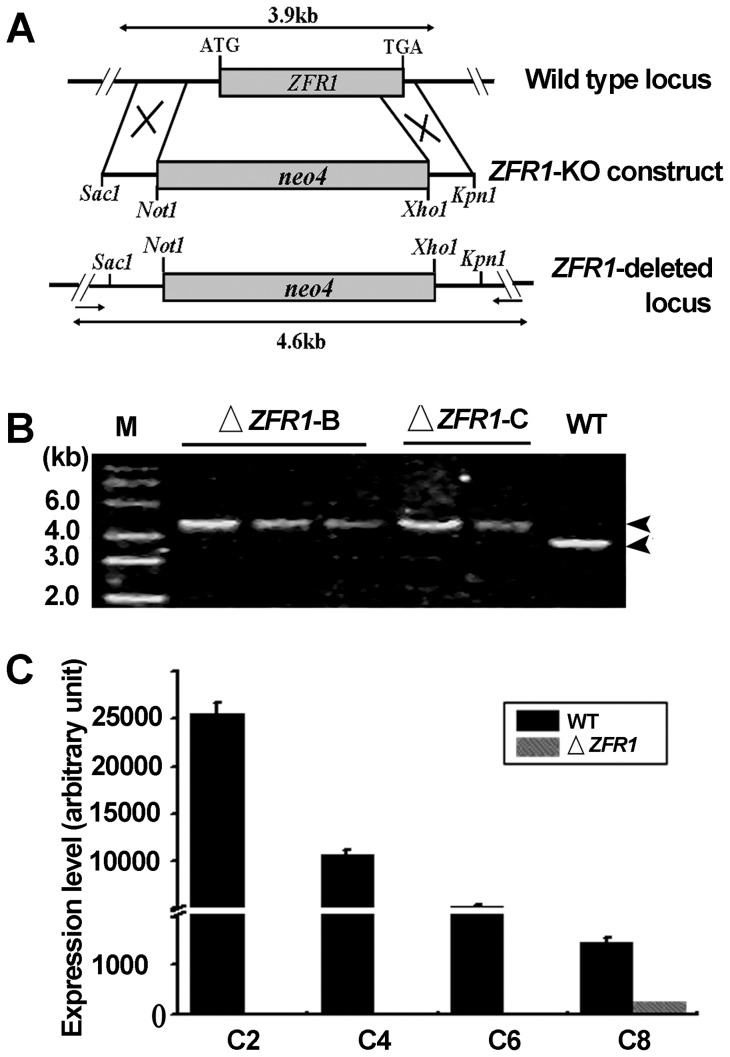
*ZFR1* is required for conjugation development. (A) Developmental profiles of wild-type cells during conjugation. Starved wild-type cells (B2086× Cu428) were mixed, and the stages of conjugation were observed by DAPI staining 2, 4, 6, 8, 10, 12, 14, 18, and 24 h after mixing. (B) Development profiles of *ZFR1* knockout cells during conjugation. Starved *ZFR1* knockout strains were mixed, and the stages of conjugation were observed by DAPI staining 2, 4, 6, 8, 10, 12, 14, 18, and 24 h after mixing. During the 7–8 h conjugation stage, there were about 20% single cells and 80% mating pairs. Unexpectedly, single cells abruptly increased to 80% during 9–10 h. (C) Developmental profiles of mating wild-type and *ZFR1* knockout cells during conjugation. Starved cells (△*ZFR1*-B2 x CU428) were mixed, and the stages of conjugation were observed by DAPI staining at 2, 4, 6, 8, 10, 12, 14, 18, and 24 h after mixing. The stages we categorized were: I, Pair Formation; II, Crescent; III, Meiosis I; IV, Meiosis II; V, 3^rd^ Prezygotic Mitosis; VI, Pronuclear Differentiation/Exchange/Fusion; VII, Postzygotic Mitosis; VIII, Macronuclear Anlagen/Nuclear Alignment; IX, Pair Separation with anlagen; X, Old macronuclear elimination; XI, Micronuclear elimination; XII, “back-out” or precocious separation associated with abortive development. For details of nuclear behavior during conjugation, see Cole et al. (1997).

To analyze Zfr1p function during conjugation, the developmental profiles of wild-type and *ZFR1* knockout cells were compared (Fig. 3A and 3B). At the initiation stage, there was no distinct difference between wild-type and *ZFR1* knockout cells. During the 7–8 h conjugation stage, about 20% of the cells were single and 80% of them had mating pairs. Unexpectedly, the proportion of single cells abruptly increased to 80% between 9–10 h. These separated single cells contained 5, 4, 3 or 2 micronuclei (Fig. S2). It seemed that the developing pairs were separated abnormally. If these separated cells were true progenies, they would have survived in the SPP medium containing 6-methylpurine. The results showed that ∼80% of the single cells were 6-methylpurine sensitive cells (Table 1). Hence, the surviving cells were not true sexual progenies. This indicated that normal development was aborted in *ZFR1* knockout cells.

**Table 1:** Viability of progenies.

Type of Mating cells	Number of cells Examined	Paromomycin Resistence Progeny	6-methylpurine Resistence Progeny
B2086×CU428	388	0 (0%)	388 (100%)
△*ZFR1*-B2×△*ZFR1*-C4	653	529 (81%)	124 (19%)
B2086×△*ZFR1*-C4	335	84 (25%)	251 (75%)

At 5–6 h post-mixing, single mating pairs were placed into drops of SPP medium and incubated for 48 h at 30°C. Completion of conjugation development was analyzed by testing for the expression of the drug resistance marker paromomycin in the parental macronuclei, or of 6-methylpurine in the newly developed macronuclei.

To confirm that developmental abortion was the real phenotype of *ZFR1* disruption, *ZFR1* knockout cells were mated with wild-type cells (Fig. 3C). Our results showed that conjugation development was rescued in these cells. It is well known that proteins can be transferred via the conjugation junctions between mating *Tetrahymena* cells [26], [29], [30]. It is possible that Zfr1p was also exchanged between wild-type cells and *ZFR1* knockout cells in our experiments. Taken together, our results suggest that somatically expressed Zfr1p is necessary for conjugation development.

### Localization of Zfr1p

**Figure 4:**
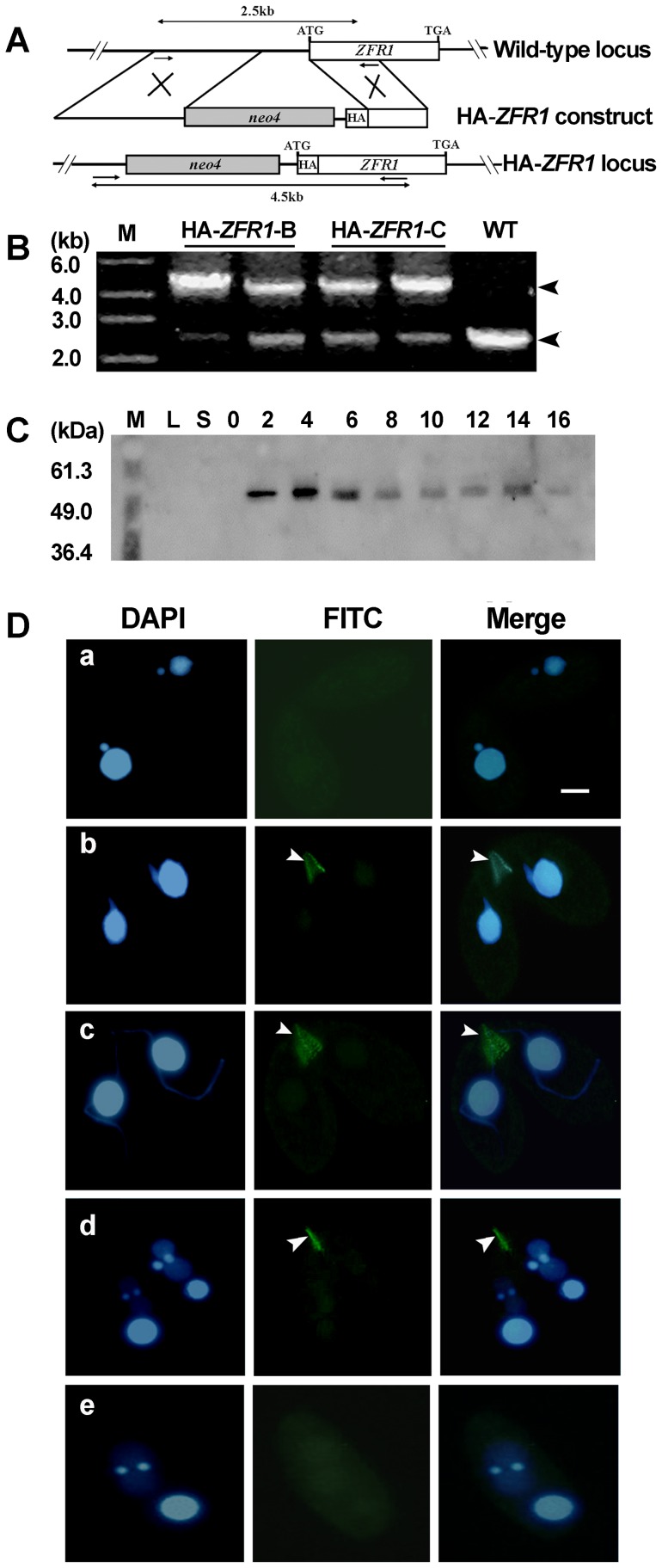
HA tagging of *ZFR1*. (A) Diagrams of the HA-*ZFR1* construct and the wild-type *ZFR1* locus. The HA epitope was inserted just after the translation start codon of *ZFR1*. The *neo4* cassette was inserted into the 5′ flanking sequence. (B) Confirmation of HA*-ZFR1* construct recombined into the sites of endogenous *ZFR1* genes. Total DNA was isolated from HA-*ZFR1* and wild-type cells. HA-*ZFR1* and *ZFR1* are shown. *ZFR1* was observed in the HA-*ZFR1* strains because the endogenous *ZFR1* gene was partially replaced. (C) Western blot analysis of HA-Zfr1p. Total cell protein was prepared from the log-phase growing, starvation, and conjugation stages (2, 4, 6, 8, 10, 12, 14, and 16 h). Total protein was separated by 12.5% SDS-PAGE and blotted onto PVDF membranes. HA-Zfr1p was probed using anti-HA monoclonal antibody. (D) Localization of HA-Zfr1p. HA-*ZFR1-B5* and HA-*ZFR1-C6* cells were mated. The cells collected at 2 hr (a and b), 4 hr (c), 8 hr (d), and 10 hr (e) post-mixing were fixed and processed for immunofluorescence staining using anti-HA primary and FITC-conjugation secondary antibodies (middle column, green). Cells were also stained with DAPI (left column). The cells were in the pair formation (a, b), crescent (c), anlagen (d), and pair separation (e) stages. Arrows indicate the conjugation junction (CJ) stained region. Scale bar, 5 µm.

To observe the localization patterns of Zfr1p, endogenous *ZFR1* was replaced with HA-*ZFR1* that encodes Zfr1p tagged with HA at the N-terminus (Fig. 4A). Endogenous *ZFR1* in the macronucleus was partially replaced by HA-*ZFR1* (Fig. 4B). HA-Zfr1p expression was examined by Western blot analysis using an anti-HA antibody (Fig. 4C). A single ∼52 kDa band was observed, consistent with the predicted molecular weight of HA-Zfr1p (52.7 kDa). The expression profile of HA-Zfr1p was consistent with the mRNA expression profile of *ZFR1*. Development of HA-*ZFR1* cells was similar to that of wild-type cells (data not shown), indicating that HA-Zfr1p was functional and reflected the function and distribution of endogenous Zfr1p.

Anti-HA staining was used to study the localization of HA-Zfr1p during the mating of different HA-*ZFR1* cell types (HA-*ZFR1*-B5 x HA-*ZFR1*-C6). Specific localization of HA-Zfr1p did not occur when the mating was initiated (Fig. 4D–a). However, during conjugation development, HA-Zfr1p was localized in the conjugation junction when micronuclei began to elongate (Fig. 4D–b). HA-Zfr1p was then constantly localized in the conjugation junction until pair separation occurred (Figs. 4D-c and 4D–d). HA-Zfr1p signal was no longer observed when the mating pairs were separated (Fig. 4D–e). Thus, HA-Zfr1p decorated the conjugation junction throughout the conjugation stage.

### Overexpression of HA-Zfr1p

**Figure 5:**
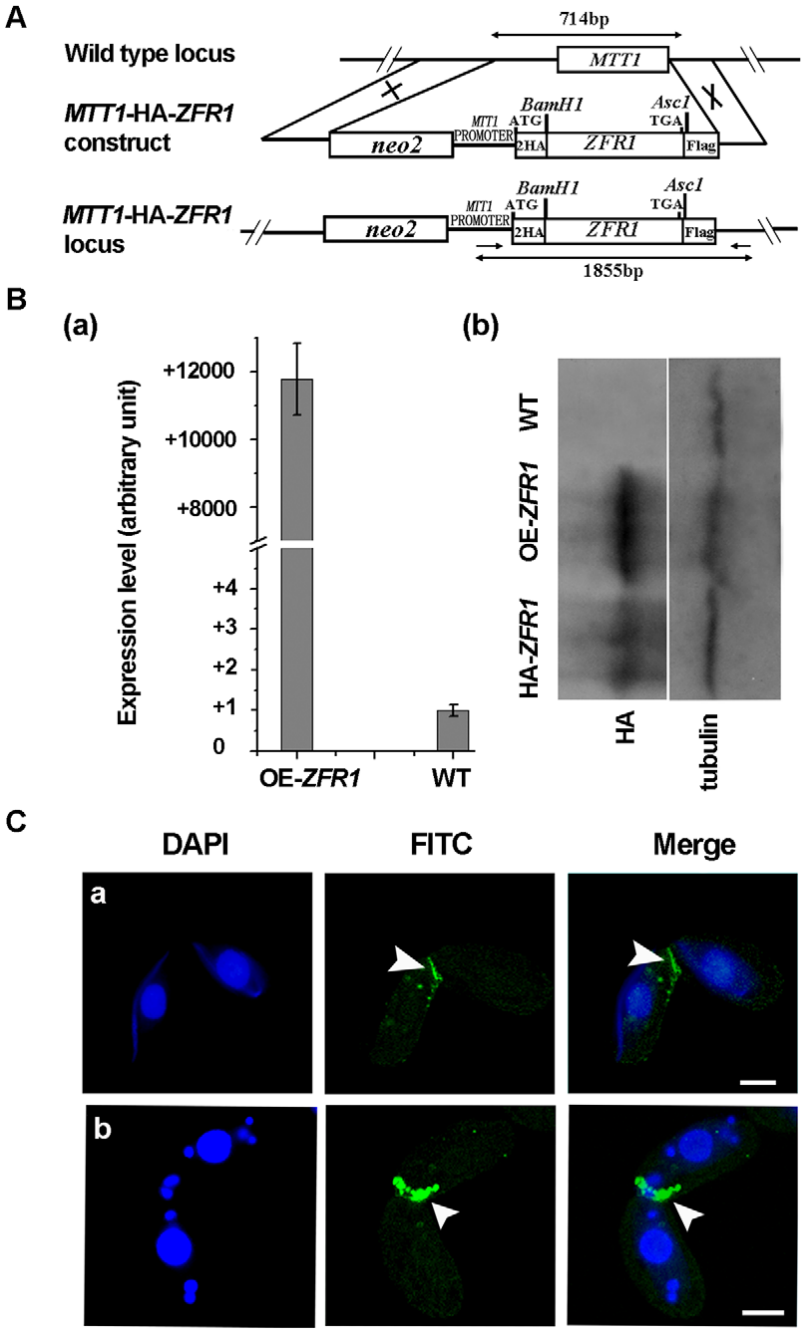
Overexpression of *ZFR1*. (A) Schematic representation of the overexpressed *ZFR1* construct. Two HA epitopes were inserted just after the initiator methionine residue. *ZFR1* digested by *BamH*1 and *Asc*1 was cloned into the plasmid pBS-HA. A *neo2* cassette was inserted into the 5′ flanking sequence of *MTT1*. (B) Analysis of *ZFR1* expression by qRT-PCR and western blotting. (a) Transcription level of HA-*ZFR1* was higher in over-expression cells. Y-axis indicates relative fluorescence strength. (b) The expression level of HA-Zfr1 was higher in over-expression cells. HA-*ZFR1* indicated expression of HA-Zfr1 under the *ZFR1* promoter. OE-*ZFR1* indicated expression of HA-Zfr1 under the *MTT1* promoter. α–tubulin was used as a loading control. (C) Localization of HA-Zfr1p. Mating pairs of *ZFR1* overexpressing cells collected at 3 and 5 h post-mixing were fixed and processed for immunofluorescence staining using anti-HA primary and FITC-conjugation secondary antibodies (middle column, green). The cells were also stained with DAPI (left column). The cells were in the crescent (a) and pronuclear exchange (b) stages. Arrows indicate the conjugation junction. *Scale bar*, 10 µm.

As described above, Zfr1p disruption resulted in the abortion of conjugation development and Zfr1p specifically decorated conjugation junctions. To further explore Zfr1p function, Zfr1p was over-expressed in *Tetrahymena* cells using an over-expression plasmid pBS-HA-*ZFR1*, in which *ZFR1* was under the control of the *MTT1* promoter (Fig. 5A). Two different mating types of over-expressed strains, namely OE-*ZFR1*-C3 and OE-*ZFR1*-B1, were obtained. Over-expression of HA-*ZFR1* was detected by qRT-PCR and western blotting (Fig. 5B). Conjugation development of cells over-expressing HA-*ZFR1* was comparable to that of wild-type cells (data not shown). Additionally, over-expressed HA-Zfr1p was specifically localized at conjugation junctions (Fig. 5C). This result further confirmed that Zfr1p specifically decorates conjugation junctions.

To understand the involvement of Zfr1p in the conjugation junction structure, a vibrating assay was performed at the early conjugation stage of mating cells. The results showed that mating pairs of the *ZFR1* knockout cells were less stable than those of wild-type cells and *ZFR1* overexpressed cells. In contrast, mating pairs of *ZFR1* overexpressed cells were more stable than those of wild-type cells (Table 2). These results indicate that Zfr1p is involved in maintaining the stability of the conjugation junction during the early stages of conjugation.

**Table 2:** Comparison of the stabilities of different conjugation cells during the early conjugation stage.

Type of Mating cells	Number of cells Examined	Average Pair Ratio Before Vibrated	Average Pair Ratio After Vibrated
OE-*ZFR1*-B1×OE-*ZFR1*-C3	4064	78.6%±5.3%	73.0%±6.1%
B2086×CU428	4028	78.4%±2.8%	70.9%±1.8%
△*ZFR1*-B2×△*ZFR1*-C4	4037	77.5%±4.4%	42.3%±10.3%

Overexpressed *ZFR1* cells (OE-*ZFR1*-C3 and OE-Z*FR1*-B1), wild-type cells (B2086 and CU428), and *ZFR1* knockout cells (△*ZFR1*-B2 and △*ZFR1*-C4) were mated. The average pair ratio before and after vibration is shown. All data were obtained from three replicates.

### Functional domain analysis of Zfr1p

**Figure 6:**
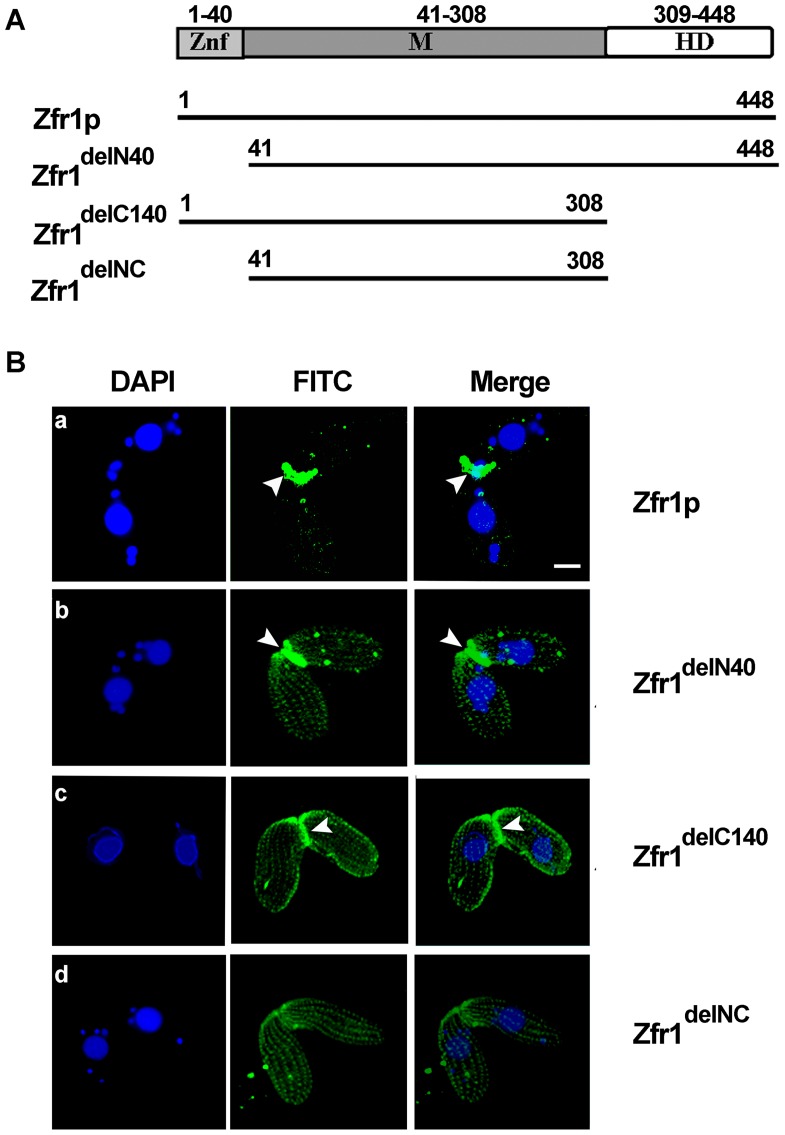
Functional domain analysis of Zfr1p. (A) Schematic representation of full-length Zfr1p and truncated Zfr1p variants: amino acids 1–40 constitute the N-terminal B-box zinc finger domain; M is the middle domain; amino acids 309–448 constitute the C-terminal hydrophobic domain. (B) Localization of HA-Zfr1, HA-Zfr1^delN40^ (*ZFR1^delN40^*-B7.1 x *ZFR1^delN40^*-C8.1), HA-Zfr1^delC140^ (*ZFR1^delC140^*-B9.2 x *ZFR1^delC140^*-C10.3), and HA-Zfr1^delCN^ (*ZFR1^delNC^*-B11.2 x *ZFR1^delNC^*-C12.5) at the conjugation stages. Mating pairs of cells were fixed and processed for immunofluorescence staining using anti-HA primary and FITC-conjugated secondary antibodies. The cells were also stained with DAPI (middle column). Arrowheads indicate the conjugation junction. Scale bar, 10 µm.

Sequence analysis showed that Zfr1p has three characteristic structural domains (Fig. 6A). To explore the function of these domains, HA-tagged constructs in which either the N-terminal B-box zinc finger domain (*ZFR1^delN40^*), or the C-terminal hydrophobic domain (*ZFR1^delC140^*), or both of these domains (*ZFR1^delNC^* ) were deleted were made. Expression of these truncated genes was under the control of the *MTT1* promoter. HA-Zfr1^delN40^ (*ZFR1^delN40^*-B7.1 x *ZFR1^delN40^*-C8.1) and HA-Zfr1^delC140^ (*ZFR1^delC140^*-B9.2 x *ZFR1^delC140^*-C10.3) were localized not only in the conjugation junction, but in linear rows of punctate foci throughout the cell (Fig. 6B–b and 6B–c). Interestingly, HA-Zfr1^delNC^ (*ZFR1^delNC^*-B11.2 x *ZFR1^delNC^*-C12.5) only formed linear rows of punctate foci throughout the mating cell (Fig. 6D). Hence, the N-terminal B-box zinc finger and C-terminal hydrophobic domains are both essential for proper localization of Zfr1p at the conjugation junction.

**Figure 7:**
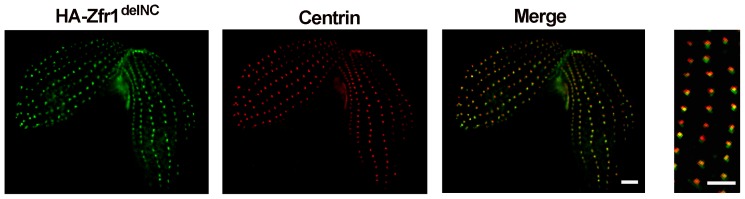
Localization of HA-Zfr1^delCN^ and Centrin. Conjugation cells expressing HA-Zfr1^delCN^ were fixed, permeabilized, and labeled with anti-HA and anti-centrin antibodies (A, B). The merged image shows the close proximity of HA-Zfr1*^delCN^* to basal bodies (C). Magnified view of the cortical region is in panel D. Scale bar, 5 µm.

**Figure 8:**
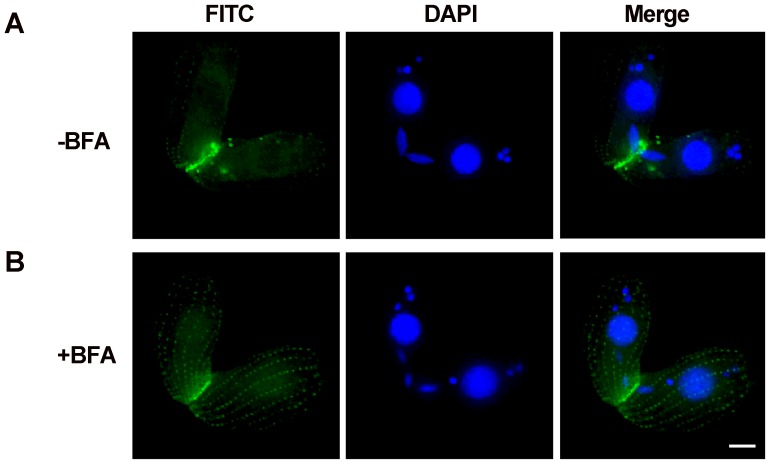
HA-Zfr1p localization in *Tetrahymena* cells with and without brefeldin A treatment. (A) Conjugation junction localization of HA-Zfr1p in untreated *Tetrahymena* cells (B) Conjugation junction localization of HA-Zfr1p decreased in cells treated with brefeldin A, and new signal decorated golgi apparatus closely associated with the mitochondria which are adjacent to cortically aligned basal body. Scale bar, 5 µm.

HA-Zfr1^delN*C*^ localized in ordered arrays near the plasma membranes of cells. This pattern was reminiscent of the arrangement of ciliary basal bodies, endosomes, Golgi apparatus and associated cortical mitochondria in *Tetrahymena* cells. To distinguish the localization of HA-Zfr1^delN*C*^, dual labeling with antibodies against HA and centrin was conducted. Our results revealed that the truncated Zfr1p protein was localized near the basal bodies of cilia (Fig. 7). Golgi apparatus and associated cortical mitochondria localize to sites near basal bodies in *Tetrahymena* [31]. To further identify HA-Zfr1^delN*C*^ location, Brefeldin A was used. Brefeldin A is known to be a potent inhibitor of protein trafficking in the endomembrane system of cells. To test whether or not HA-Zfr1p trafficking is dependent on the Golgi apparatus, we compared HA-Zfr1p localization in cells treated with Brefeldin A and untreated cells. Brefeldin A treatment did indeed disrupt HA-Zfr1p localization at the conjugation junction in these cells, resulting in localization in ordered arrays near the plasma membrane (Fig. 8). This localization pattern of Zfr1p was similar to the localization of truncated Zfr1p.

## Discussion

The model eukaryote *T. thermophila* is the first ciliated protozoan whose genome has been sequenced and whose genome-wide expression profile has been catalogued [18], [19]. From the Macronuclear genome of this organism and subsequent analysis through comparative genomic hybridization, 24,725 protein-coding genes have been predicted [32]. Based on published microarray data for *T. thermophilia*, 1068 genes were identified, which are specifically expressed during the conjugation stage [19]. There are clusters of genes that exhibit distinct patterns of expression, which can be used to identify candidate genes involved in the striking developmental changes that occur during conjugation [19]. *TWI1*, which encodes an essential argonaute family Twi1p that is required for IES sequences elimination [26]. Four proteins (CnjBp, Wag1p, Ema1p and Giw1p) have been shown to be physically associated with Twi1p by co-immunoprecipitation and TAP-tagging [33]–[35]. Twi1p was also identified from isolated exchange junction preparation [12]. Similarly, the signal was detected at the junction of cells using anti-Ema1p antiserum [34]. So, Ema1p and Twi1p not only function in DNA elimination, they could also be involved in function of conjugation junction. In the study, we sought to identify new candidate functional genes associated with the conjugation junction in *Tetrahymena*.

*ZFR1* is specifically expressed at conjugation stage. The expression patterns of *ZFR1* imply Zfr1p play an important role in the stage. Immunofluorescence staining showed Zfr1p specifically localized at the conjugation junction. Although Zfr1p has no identified homolog in other organisms, it includes a zinc finger structure and a hydrophobic C-terminal domain. Zinc finger domains were first identified as DNA-binding motifs in the transcription factor TFIIIA in *Xenopus laevis* [36]. Now, they are recognized to bind DNA, RNA, protein and/or lipid substrates [37]. Zinc finger-containing proteins function in gene transcription, translation, mRNA trafficking, cytoskeleton organization, zinc sensing, protein folding, chromatin remodeling, and cell adhesion [15]. In some cases, zinc fingers have been found to be crucial for the subcellular localization of proteins. For instance, the GTPase-activating protein ARF1 is localized to the Golgi complex via its zinc finger-like domains [38] and the autoantigen EEA1 is localized to endosomes via its zinc-binding FYVE finger [39]. In the present study, we found that the zinc finger domain of Zfr1p was required for Zfr1p specific localization at conjugation junctions. Truncated Zfr1p^delN40^ localized not only in the conjugation junction, but decorated Golgi apparatus.

It is well known that conjugation junction formation involves intricate interactions between membrane proteins, cytoskeletal frameworks, and lipids [8]. The cytoplasm near the conjugation junction contains numerous assorted membrane structures. These structures include lamellae, tubes, and small vesicles. Cda13p, containing membrane-spanning domains, is associated with the Golgi apparatus. When GFP-Cda13p was expressed in mating cells, a transient pattern of localization in which the fusion protein appeared to decorate a ring associated with the nuclear exchange junction was observed. Further studies have shown that Cda13p participates in events associated with remodeling of the nuclear exchange junction during *Tetrahymena* conjugation stage [14]. In the study, we found C-terminal hydrophobic domain of Zfr1p was also necessary for its specific conjugation junction localization. Furthermore, when the N-terminal zinc finger and C-terminal hydrophobic domains of Zfr1p were disrupted, localization of Zfr1^delNC^ at conjugation junctions completely disappeared. So, it is clear that the C-terminal hydrophobic and N-terminal zinc finger domains of Zfr1p mediate interactions between Zfr1p and conjugation junction membrane structures or proteins. Accompanying truncated Zfr1^delNC^ disappeared at the conjugation junction, it occured at cortical puncta in longitudinal rows. It is well known that basal body, mitochondria, Golgi apparatus and endosomes are arranged in longitudinal rows in *Tetrahymena* [14], [20], [31]. Based on their different function, the Golgi apparatus is integral in modifying, sorting, and packaging macromolecules for secretion or use within the cell. To explore the relation between Zfr1p and Golgi apparatus, Brefeldin A, which inhibits protein trafficking in the endomembrane system of cells, was used. We found HA-Zfr1p localization in cells treated with Brefeldin A to be similar to the localization of truncated Zfr1p in untreated cells. Taken together, our results suggest that Zfr1p is transferred to conjugation junctions by Golgi apparatus and decorates the conjugation junctions via the N-terminal B-box zinc finger and C-terminal hydrophobic domains.

Originally, 15 proteins were identified from exchange junction preparations isolated via ethanol fixation and sonication. Using the method, many membrane proteins and proteins that are only loosely associated with the nuclear exchange junctions were lost. In addition, the identified 15 proteins were not conjugation junction specific. Fenestrin was seen over the oral apparatus, oral primordium, and the base of each cilium within the longitudinal ciliary rows and nuclear exchange junction [12]. Recently, it has been found that β-tubulin multigene family member BLT1 participated in formation of the microtubules of the meiotic apparatus of the micronucleus during conjugation. BLT1 also decorated conjugation junction [40]. Ema1p localizes in old and new Macs during conjugation. Conjugation junction was also observed using anti-Ema1p antiserum [34]. GFP-Cda13p was localized along cortical rows and in cytoplasmic puncta. When GFP-Cda13p was expressed in mating cells, it also decorated a ring associated with the nuclear exchange junction [14]. So far, we found Zfr1p was only protein which specifically localized on the conjugation junction. So, we think more unidentified conjugation junction specific factors remain to explore. Although co-expressed genes in TGED and the network in TFGD are unavoidable to have some false positives, they are indeed useful to identify genes with related functions [19]. Based on the similar expression pattern and functional domains, four *ZFR1* co-expressed genes, *TLR1, TDT1, ZFR2* and *ZFR3*, were also identified using the TGED database and the Tetrahymena gene network (TGN) [19], [28]. When *ZFR2* or *ZFR3* was knocked out, mating cells showed abnormal development phenotype which is similar to that of *ZFR1* (data not shown). These results imply that these candidated co-expressed genes could be involved in the Zfr1p conjugation junction signal pathway. The physical and functional interaction of these co-expressed genes warrants further investigation.

Development of mating *ZFR1* knockout cells looked normal at early stages. However, the conjugation junction structures between *ZFR1* knockout cells were less stable than those of wild-type and *ZFR1* over-expressing cells. These results implied the involvement of Zfr1p in conjugation junction structure stability. Importantly, an elaborate conjugation junction is required for pronuclear exchange during the conjugation stage in *Tetrahymena* [13]. The process of pronuclear exchange involves dramatic membrane remodeling to accompany the formation and resolution of the nuclear exchange junction during mating [14]. Evidence of abnormal development of *ZFR1* knockout cells appeared 8 h into the conjugation stage, and true sexual progenies could not be produced. The separated single cells contained abnormal number of micronucleus (Fig. S2). These results indicate that pronuclear exchange or conjugation junction remodeling was aborted in *ZFR1* knockout cells, which lead to abortion of sexual development.

Taken together, our study showed that *ZFR1* is conjugation specific expressed protein. Zfr1p is first identified protein which only localized on the conjugation junction. It is essential for the sexual life cycle of *Tetrahymena* cells. Further studies on the interaction network of this protein will help to understand the highly complex and ordered restructuring of the conjugation junction structure in *Tetrahymena*.

## Supporting Information

Figure S1 **Micrroarray data of** ***ZFR1*** **and candidate co-expressed gene.** Microarray data (TFGD, http://tfgd.ihb.ac.cn/search/detail/gene) showing gene expression patterns of *ZFR1* gene and five candidate co-expressed genes *EZL1, TDT1, TLR1, ZFR2* and *ZFR3* during the three physiological stages: vegetative growth ([L-1] low cell density [100,000 cells per milliliter]; [L–m] medium density [350,000 cells per milliliter]; [L–h] high cell density [1,000,000 cells per milliliter], starvation ([S-0] 0 h; [S-3] 3 h; [S-6] 6 h; [S-9] 9 h; [S-12] 12 h; [S-15] 15 h; [S-24] 24 h), and conjugation ([C-0] 0 h; [C-2] 2 h; [C-4] 4 h; [C-6] 6 h; [C-8] 8 h; [C-10] 10 h; [C-12] 12 h; [C-14] 14 h; [C-16] 16 h; [C-18] 18 h).
(TIF)

Figure S2 **The development profile of the nuclei in the knockout** ***ZFR1*** **mating cells.** (A) The normal developmental nuclei. After 7–8 h postmixing, only 20% pairs could complete development (a–j). (B) The single cells which precocious associated with abortive development or “back-out” cells. About 80% pairs miscarried after 7–8 h postmixing (k–o). The nuclei were observed by DAPI staining at 2, 4, 6, 8, 10, 12, 14, 18, and 24 h after mixing. Arrows indicate micronuclei. *Scale bar*, 10 um.
(TIF)

Table S1 **Primers used in the present study.**
(DOC)

Table S2 **Genotypes and phenotypes of** ***T. thermophila*** **strains used in the present study.**
(DOC)

## References

[1] Green KJ, Getsios S, Troyanovsky S, Godsel LM (2010) Intercellular junction assembly, dynamics, and homeostasis. Cold Spring Harb Perspect Biol 2: a000125. 20182611 10.1101/cshperspect.a000125 PMC2828282

[2] Pinto da Silva P, Kachar B (1982) On tight-junction structure. Cell 28: 441–450. 6804093 10.1016/0092-8674(82)90198-2

[3] Goodenough DA, Paul DL (2009) Gap junctions. Cold Spring Harb Perspect Biol 1: a002576. 20066080 10.1101/cshperspect.a002576 PMC2742079

[4] Beyer EC, Paul DL, Goodenough DA (1990) Connexin family of gap junction proteins. J Membr Biol 116: 187–194. 2167375 10.1007/BF01868459

[5] Juliano RL (2002) Signal transduction by cell adhesion receptors and the cytoskeleton: functions of integrins, cadherins, selectins, and immunoglobulin-superfamily members. Annu Rev Pharmacol Toxicol 42: 283–323. 11807174 10.1146/annurev.pharmtox.42.090401.151133

[6] Sugiura M, Harumoto T (2001) Identification, characterization, and complete amino acid sequence of the conjugation-inducing glycoprotein (blepharmone) in the ciliate Blepharisma japonicum. Proc Natl Acad Sci U S A 98: 14446–14451. 11724922 10.1073/pnas.221457698 PMC64701

[7] Cole ES, Soelter TA (1997) A mutational analysis of conjugation in Tetrahymena thermophila. 2. Phenotypes affecting middle and late development: third prezygotic nuclear division, pronuclear exchange, pronuclear fusion, and postzygotic development. Dev Biol 189: 233–245. 9299116 10.1006/dbio.1997.8649

[8] Ostrowski SG, Van Bell CT, Winograd N, Ewing AG (2004) Mass spectrometric imaging of highly curved membranes during Tetrahymena mating. Science 305: 71–73. 15232100 10.1126/science.1099791 PMC2833272

[9] Suganuma Y, Shimode C, Yamamoto H (1984) Conjugation in Tetrahymena: formation of a special junction area for conjugation during the co-stimulation period. J Electron Microsc (Tokyo) 33: 10–18. 6491567

[10] Orias JD, Hamilton EP, Orias E (1983) A microtubule meshwork associated with gametic pronucleus transfer across a cell-cell junction. Science 222: 181–184. 6623070 10.1126/science.6623070

[11] Wolfe J (1982) The conjugation junction of Tetrahymena: its structure and development. Journal of Morphology 172: 159–178. 10.1002/jmor.1051720204 30096979

[12] Cole ES, Anderson PC, Fulton RB, Majerus ME, Rooney MG, et al (2008) A proteomics approach to cloning fenestrin from the nuclear exchange junction of Tetrahymena. J Eukaryot Microbiol 55: 245–256. 18681839 10.1111/j.1550-7408.2008.00337.x

[13] Gaertig J, Cole ES (2000) The role of cortical geometry in the nuclear development of Tetrahymena thermophila. J Eukaryot Microbiol 47: 590–596. 11128713 10.1111/j.1550-7408.2000.tb00095.x

[14] Zweifel E, Smith J, Romero D, Giddings TH, Winey M, et al (2009) Nested genes CDA12 and CDA13 encode proteins associated with membrane trafficking in the ciliate Tetrahymena thermophila. Eukaryot Cell 8: 899–912. 19286988 10.1128/EC.00342-08 PMC2698308

[15] Laity JH, Lee BM, Wright PE (2001) Zinc finger proteins: new insights into structural and functional diversity. Curr Opin Struct Biol 11: 39–46. 11179890 10.1016/s0959-440x(00)00167-6

[16] Chiu CF, Ghanekar Y, Frost L, Diao A, Morrison D, et al (2008) ZFPL1, a novel ring finger protein required for cis-Golgi integrity and efficient ER-to-Golgi transport. EMBO J 27: 934–947. 18323775 10.1038/emboj.2008.40 PMC2323254

[17] Gleason EJ, Lindsey WC, Kroft TL, Singson AW, L'Hernault SW (2006) spe-10 encodes a DHHC-CRD zinc-finger membrane protein required for endoplasmic reticulum/Golgi membrane morphogenesis during Caenorhabditis elegans spermatogenesis. Genetics 172: 145–158. 16143610 10.1534/genetics.105.047340 PMC1456142

[18] Eisen JA, Coyne RS, Wu M, Wu D, Thiagarajan M, et al (2006) Macronuclear genome sequence of the ciliate Tetrahymena thermophila, a model eukaryote. PLoS Biol 4: e286. 16933976 10.1371/journal.pbio.0040286 PMC1557398

[19] Miao W, Xiong J, Bowen J, Wang W, Liu Y, et al (2009) Microarray analyses of gene expression during the Tetrahymena thermophila life cycle. PLoS ONE 4: e4429. 19204800 10.1371/journal.pone.0004429 PMC2636879

[20] Kurz S, Tiedtke A (1993) The Golgi apparatus of Tetrahymena thermophila. J Eukaryot Microbiol 40: 10–13. 8457796 10.1111/j.1550-7408.1993.tb04874.x

[21] Gorovsky MA, Yao MC, Keevert JB, Pleger GL (1975) Isolation of micro- and macronuclei of Tetrahymena pyriformis. Methods Cell Biol 9: 311–327. 805898 10.1016/s0091-679x(08)60080-1

[22] Allis CD, Dennison DK (1982) Identification and purification of young macronuclear anlagen from conjugating cells of Tetrahymena thermophila. Dev Biol 93: 519–533. 7141113 10.1016/0012-1606(82)90139-7

[23] Liu Y, Taverna SD, Muratore TL, Shabanowitz J, Hunt DF, et al (2007) RNAi-dependent H3K27 methylation is required for heterochromatin formation and DNA elimination in Tetrahymena. Genes Dev 21: 1530–1545. 17575054 10.1101/gad.1544207 PMC1891430

[24] Cassidy-Hanley D, Bowen J, Lee JH, Cole E, VerPlank LA, et al (1997) Germline and somatic transformation of mating Tetrahymena thermophila by particle bombardment. Genetics 146: 135–147. 9136007 10.1093/genetics/146.1.135 PMC1207932

[25] Coyne RS, Nikiforov MA, Smothers JF, Allis CD, Yao MC (1999) Parental expression of the chromodomain protein Pdd1p is required for completion of programmed DNA elimination and nuclear differentiation. Mol Cell 4: 865–872. 10619033 10.1016/s1097-2765(00)80396-2

[26] Mochizuki K, Fine NA, Fujisawa T, Gorovsky MA (2002) Analysis of a piwi-related gene implicates small RNAs in genome rearrangement in tetrahymena. Cell 110: 689–699. 12297043 10.1016/s0092-8674(02)00909-1

[27] Mochizuki K (2008) High efficiency transformation of Tetrahymena using a codon-optimized neomycin resistance gene. Gene 425: 79–83. 18775482 10.1016/j.gene.2008.08.007

[28] Xiong J, Yuan D, Fillingham JS, Garg J, Lu X, et al (2011) Gene network landscape of the ciliate Tetrahymena thermophila. PLoS ONE 6: e20124. 21637855 10.1371/journal.pone.0020124 PMC3102692

[29] McDonald BB (1966) The exchange of RNA and protein during conjugation in Tetrahymena. J Protozool 13: 277–285. 5953847 10.1111/j.1550-7408.1966.tb01908.x

[30] Yin L, Gater ST, Karrer KM (2010) A developmentally regulated gene, ASI2, is required for endocycling in the macronuclear anlagen of Tetrahymena. Eukaryot Cell 9: 1343–1353. 20656911 10.1128/EC.00089-10 PMC2937337

[31] Elde NC, Morgan G, Winey M, Sperling L, Turkewitz AP (2005) Elucidation of clathrin-mediated endocytosis in tetrahymena reveals an evolutionarily convergent recruitment of dynamin. PLoS Genet 1: e52. 16276403 10.1371/journal.pgen.0010052 PMC1277907

[32] Coyne RS, Thiagarajan M, Jones KM, Wortman JR, Tallon LJ, et al (2008) Refined annotation and assembly of the Tetrahymena thermophila genome sequence through EST analysis, comparative genomic hybridization, and targeted gap closure. BMC Genomics 9: 562. 19036158 10.1186/1471-2164-9-562 PMC2612030

[33] Bednenko J, Noto T, DeSouza LV, Siu KW, Pearlman RE, et al (2009) Two GW repeat proteins interact with Tetrahymena thermophila argonaute and promote genome rearrangement. Mol Cell Biol 29: 5020–5030. 19596782 10.1128/MCB.00076-09 PMC2738283

[34] Aronica L, Bednenko J, Noto T, DeSouza LV, Siu KW, et al (2008) Study of an RNA helicase implicates small RNA-noncoding RNA interactions in programmed DNA elimination in Tetrahymena. Genes Dev 22: 2228–2241. 18708581 10.1101/gad.481908 PMC2518816

[35] Noto T, Kurth HM, Kataoka K, Aronica L, DeSouza LV, et al (2010) The Tetrahymena argonaute-binding protein Giw1p directs a mature argonaute-siRNA complex to the nucleus. Cell 140: 692–703. 20211138 10.1016/j.cell.2010.02.010 PMC2845462

[36] Shastry BS, Honda BM, Roeder RG (1984) Altered levels of a 5 S gene-specific transcription factor (TFIIIA) during oogenesis and embryonic development of Xenopus laevis. J Biol Chem 259: 11373–11382. 6206067

[37] Klug A (2010) The discovery of zinc fingers and their development for practical applications in gene regulation and genome manipulation. Q Rev Biophys 43: 1–21. 20478078 10.1017/S0033583510000089

[38] Cukierman E, Huber I, Rotman M, Cassel D (1995) The ARF1 GTPase-activating protein: zinc finger motif and Golgi complex localization. Science 270: 1999–2002. 8533093 10.1126/science.270.5244.1999

[39] Stenmark H, Aasland R, Toh BH, D'Arrigo A (1996) Endosomal localization of the autoantigen EEA1 is mediated by a zinc-binding FYVE finger. J Biol Chem 271: 24048–24054. 8798641 10.1074/jbc.271.39.24048

[40] Pucciarelli S, Ballarini P, Sparvoli D, Barchetta S, Yu T, et al (2012) Distinct functional roles of beta-tubulin isotypes in microtubule arrays of Tetrahymena thermophila, a model single-celled organism. PLoS ONE 7: e39694. 22745812 10.1371/journal.pone.0039694 PMC3382179

