# Supplementary figures and images for: The Zinc Finger Protein Zfr1p Is Localized Specifically to Conjugation Junction and Required for Sexual Development in Tetrahymena thermophila
Source: PLoS One. 2012 Dec 10;7(12):e52799. doi: 10.1371/journal.pone.0052799 (PMC3519685; doi:10.1371/journal.pone.0052799)

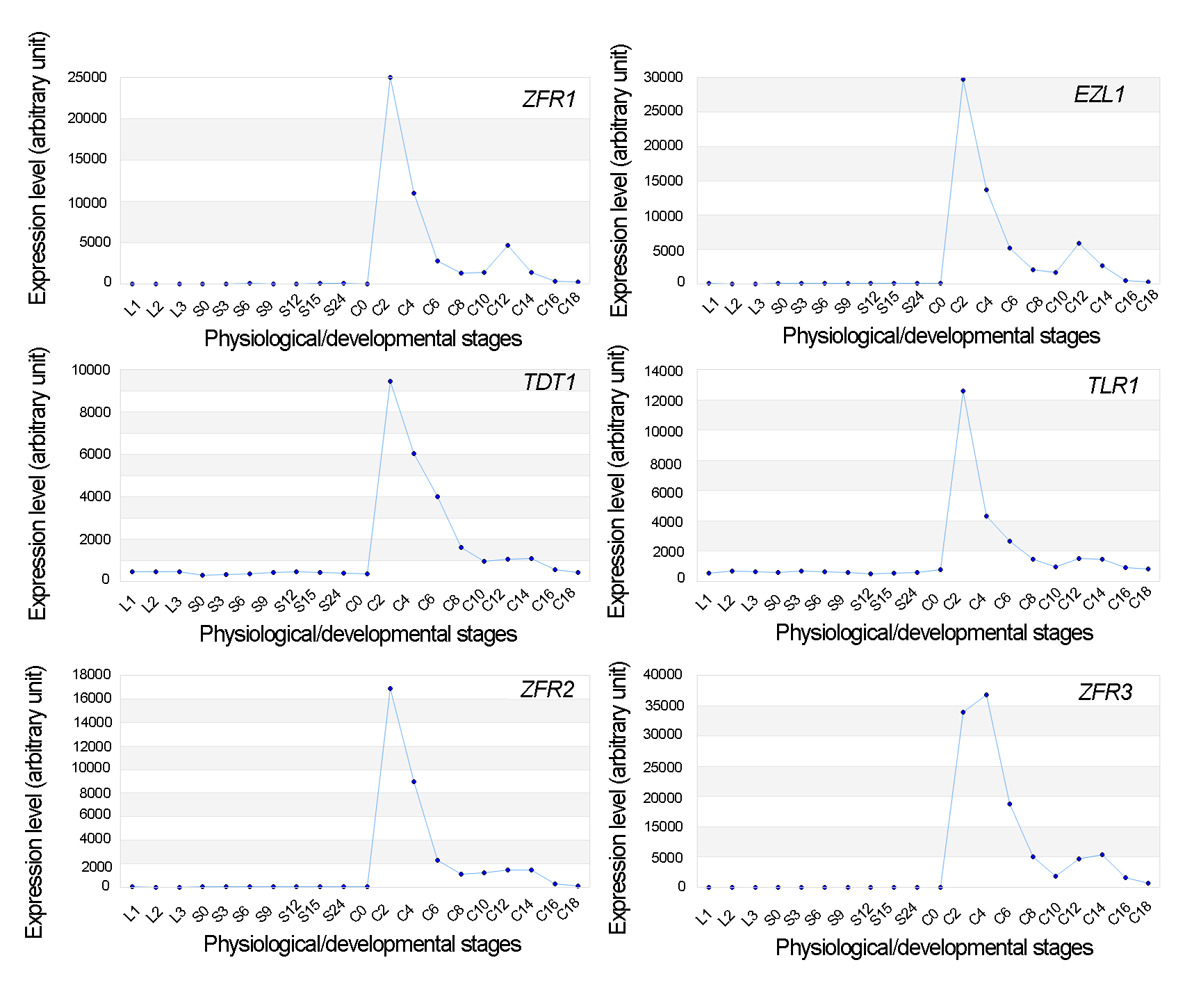

Supplement: Figure S1 — Micrroarray data of ZFR1 and candidate co-expressed gene. Microarray data (TFGD, http://tfgd.ihb.ac.cn/search/detail/gene) showing gene expression patterns of ZFR1 gene and five candidate co-expressed genes EZL1, TDT1, TLR1, ZFR2 and ZFR3 during the three physiological stages: vegetative growth ([L-1] low cell density [100,000 cells per milliliter]; [L–m] medium density [350,000 cells per milliliter]; [L–h] high cell density [1,000,000 cells per milliliter], starvation ([S-0] 0 h; [S-3] 3 h; [S-6] 6 h; [S-9] 9 h; [S-12] 12 h; [S-15] 15 h; [S-24] 24 h), and conjugation ([C-0] 0 h; [C-2] 2 h; [C-4] 4 h; [C-6] 6 h; [C-8] 8 h; [C-10] 10 h; [C-12] 12 h; [C-14] 14 h; [C-16] 16 h; [C-18] 18 h). (TIF) [file pone.0052799.s001.tif]

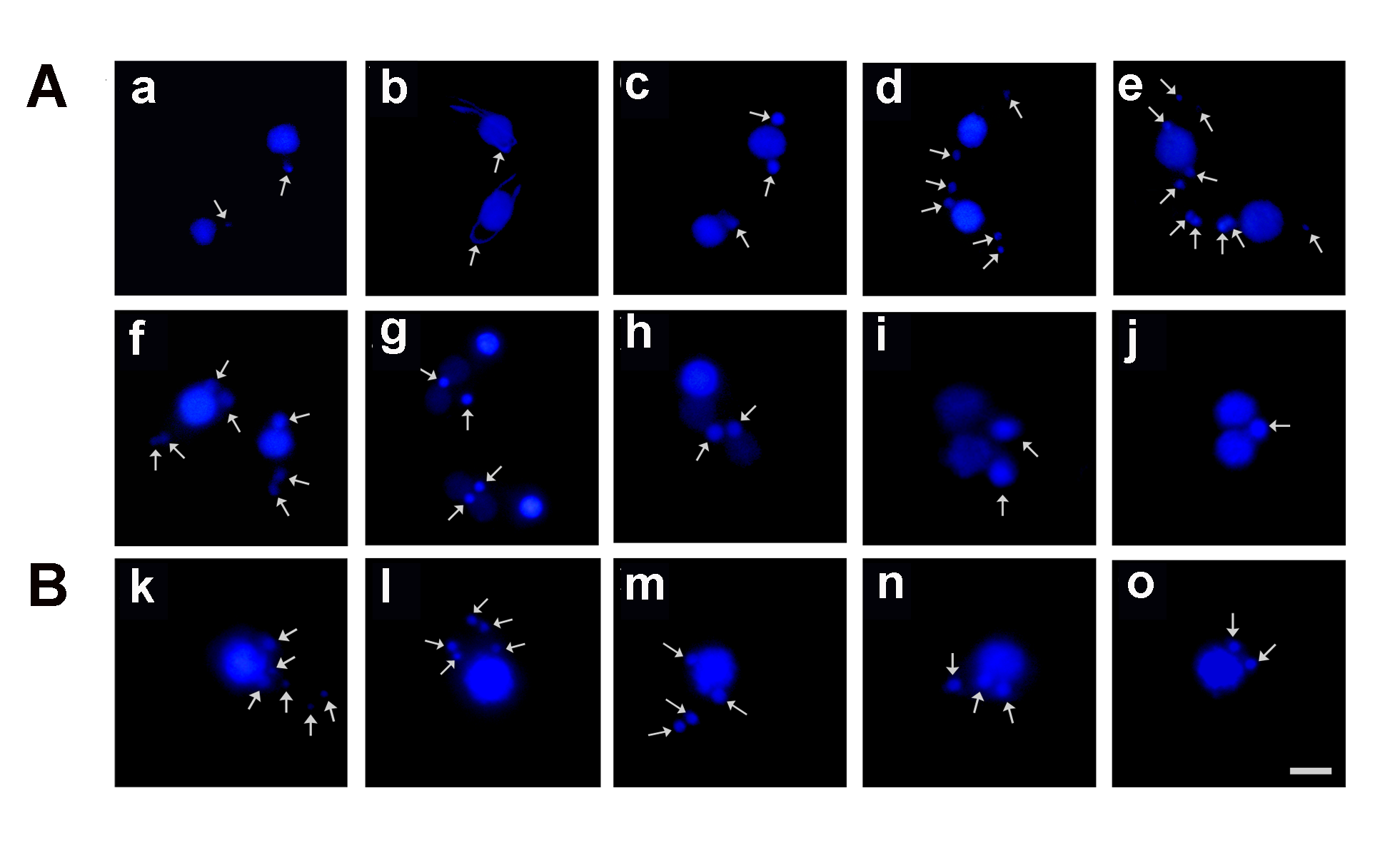

Supplement: Figure S2 — The development profile of the nuclei in the knockout ZFR1 mating cells. (A) The normal developmental nuclei. After 7–8 h postmixing, only 20% pairs could complete development (a–j). (B) The single cells which precocious associated with abortive development or “back-out” cells. About 80% pairs miscarried after 7–8 h postmixing (k–o). The nuclei were observed by DAPI staining at 2, 4, 6, 8, 10, 12, 14, 18, and 24 h after mixing. Arrows indicate micronuclei. Scale bar, 10 um. (TIF) [file pone.0052799.s002.tif]
