# Supplementary material for: The Zinc Finger Protein Zfr1p Is Localized Specifically to Conjugation Junction and Required for Sexual Development in Tetrahymena thermophila
Source: PLoS One. 2012 Dec 10;7(12):e52799. doi: 10.1371/journal.pone.0052799 (PMC3519685; doi:10.1371/journal.pone.0052799)
Supplement: Table S1 — Primers used in the present study. (DOC) [file pone.0052799.s003.doc]

| Primer name | Sequence |
| --- | --- |
| KO-5'FW | TGTTGTTTGGAGTTTTCTTACTTACTT |
| KO-5'RV | TCCTGCAGCACCTGAAAATATCTTAGCTAGCTA |
| KO-3'FW | CCACTAGTTCTAATAAAGGAGCATCACAATAAATAGC |
| KO-3'RV | TGCCAAGCAAGCAGGAAATGTAAAAT |
| *neo4*FW | AAAAATTAGTTTATTAGCTAGCTAAGATATTTTCAGGTGCTGCAGGAATTCGATAGACAA |
| *neo4*RV | CCTTAAAATGCCAAGCTATTTATTGTGATGCTCCTTTATTGGCCGCTCTAGAACTAGTGG |
| KO-FW | GAAAAGTATGACAAAATTAACTGAAAAGGA |
| KO-RV | GAAAGTAGGAAAAAATTAATCACTGTTT |
| *ZFR1*-Fi | TGATGACCATCTATTTATCTCAAACTG |
| *ZFR1*-Ri | TTCCAGAAGCATACTATTGGATAAACT |
| qRT-Pup | CTGAGCAGTTCAAGAAATATCGCTC |
| qRT-Pdown | CGTCTAACTCTTCAATTTATGGGCC |
| 17s-FW | GGTTCGACTTAGGGAGTAAACATTT |
| 17s-RV | AAATGTTTACTCCCTAAGTCGAACC |
| HA-5'FW | AGCATCCAAACAAAATTTTAATTGG |
| HA-5'RV | CATATGGATAAGCATAATCAGGAACATCATAAGGATACATTTTATTTTTCAGATTATTTACAAA |
| HA-3'FW | CCTGATTATGCTTATCCATATGATGTTCCTGATTATGCTAGTTAAATCTGTAGTATTCATTAATCTG |
| HA-3'RV | ATTCTTTCTTGTTATTAGGCTAGAATCT |
| HA-*neo4*FW | TCAAATTTTTACTGGAAAAATGCAGAAGGAAGAACTAACTATTCTTTAAG |
| HA-NextRV | TTTTTCCAATTGATTCTGTCTGTTT |
| HA-*neo4*FW | TGTTTTTAATTTTATTGAATAACTCCTTTAATTTAAATACAC |
| HA-*neo4*RV | AGTTAGTTCTTCCTTCTGCATTTTTCCAGTAAAAATTTGA |
| *neo4*-5'-FW | TGATGACCATCTATTTATCTCAAACTG |
| *neo4*-5'-RV | GTGTATTTAAATTAAAGGAGTTATTCATTCCAAATTTATGCTTCATAAGTCATTG |
| HA-FW | TGTTGTTTGGAGTTTTCTTACTTACTT |
| HA-RV | AACTTTTTTGCTGAGAAATATGGCTTT |
| MTT1-FW | GCTACGTGATTCACGATTTATGCAATG |
| MTT1-RV | CGAAACTGATTTTATGCAATTATGAATTAC |
| OE-FW | ACGGATCCAGTTAAATCTGTAGTATTCATTAATC |
| OE-RV | ATGGCGCGCCTCAATTAAAGTCAATCAATTCATATA |
| truncated-5'-FW | GACGGATCCATAATCCCTTTTTAGGAGTTTTGT |
| truncated-5'-RV | ATGGCGCGCCTCAATTAAAGTCAATCAATTCATATA |
| truncated-3'-FW | ACGGATCCAGTTAAATCTGTAGTATTCATTAATC |
| truncated-3'-RV | ATGGCGCGCCTCAACTTTGAAATTCTTTCTTGTTATTAGGCT |
| 5'BamH1-T-FW | ACGGATCCATAAAGGCTAGTTAAACCTAAAGCGAG |
| 3'Asc1-T-RV | ATGGCGCGCCTCAACTTTGAAATTCTTTCTTGTTATTAGGCT |
