# Supplementary material for: The Zinc Finger Protein Zfr1p Is Localized Specifically to Conjugation Junction and Required for Sexual Development in Tetrahymena thermophila
Source: PLoS One. 2012 Dec 10;7(12):e52799. doi: 10.1371/journal.pone.0052799 (PMC3519685; doi:10.1371/journal.pone.0052799)
Supplement: Table S2 — Genotypes and phenotypes of T. thermophila strains used in the present study. (DOC) [file pone.0052799.s004.doc]

| **Strain** | **Micronuclear genotype** | **Macronuclear genotype** | **Phenotype** |
| --- | --- | --- | --- |
| CU428 | *mpr-1/mpr-1* | Wild type | mp-s, VII |
| B2086 | Wild type | Wild type | cy-s, mp-s, II |
| △*ZFR*-1-C4 | *mpr-1/mpr-1* | *ZFR1::neo4, ZFR1::neo4* | pm-r, mp-s, VII |
| △*ZFR*-1-B2 | Wild type | *ZFR1::neo4, ZFR1::neo4* | pm-r, mp-s, II |
| HA-*ZFR1*-C6 | *mpr-1/mpr-1* | *ZFR1, ZFR1HA::neo4* | pm-r, mp-s, VII |
| HA-*ZFR1*-B5 | Wild type | *ZFR1, ZFR1HA::neo4* | pm-r, mp-s, II |
| OE-*ZFR1*-C3 | *mpr-1/mpr-1* | *MTT1, ZFR1HA::neo4* | pm-r, mp-s, VII |
| OE-*ZFR1*-B1 | Wild type | *MTT1, ZFR1HA::neo4* | pm-r, mp-s, II |
| *ZFR1^delN40^*-C8.1 | *mpr-1/mpr-1* | *MTT1, ZFR1HA::neo4* | pm-r, mp-s, VII |
| *ZFR1^delN40^*-B7.1 | Wild type | *MTT1, ZFR1HA::neo4* | pm-r, mp-s, II |
| *ZFR1^delc140^*-C10.3 | *mpr-1/mpr-1* | *MTT1, ZFR1HA::neo4* | pm-r, mp-s, VII |
| *ZFR1^delc140^*-B9.2 | Wild type | *MTT1, ZFR1HA::neo4* | pm-r, mp-s, II |
| *ZFR1^delNC^*-C12.5 | *mpr-1/mpr-1* | *MTT1, ZFR1HA::neo4* | pm-r, mp-s, VII |
| *ZFR1^delNC^*-C11.2 | Wild type | *MTT1, ZFR1HA::neo4* | pm-r, mp-s, II |
